# Reducing the Negative Environmental Impact of Winter Airport Maintenance through Its Model Design and Simulation

**DOI:** 10.3390/ijerph17041296

**Published:** 2020-02-18

**Authors:** Peter Koščák, Štefan Berežný, Iveta Vajdová, Ivan Koblen, Mateusz Ojciec, Darina Matisková, Tomáš Puškáš

**Affiliations:** 1 Department of Air Transport Management, Faculty of Aeronautics, Technical University of Kosice, Rampová 7, 04121 Kosice, Slovakia; peter.koscak@tuke.sk (P.K.); puskas.tomas@gmail.com (T.P.); 2 Department of Mathematics and Theoretical Informatics, Faculty of Electrical Engineering and Informatics, Technical university of Kosice, Letná 9, 04001 Kosice, Slovakia; stefan.berezny@tuke.sk; 3 Transport Authority SR, Civil Aviation Division, Airworthiness Division, M.R. Štefánik Airport, 823 05 Bratislava, Slovakia; ivan.koblen@nsat.sk; 4 Department of Aviation Engineering, Faculty of Aeronautics, Technical University of Kosice, Rampová 7, 04121 Kosice, Slovakia; mateusz.ojciec92@gmail.com; 5 Faculty of Manufacturing Technologies, Technical University of Kosice, Bayerova 1, 080 01 Prešov, Slovakia; darina.matiskova@tuke.sk

**Keywords:** airport, algorithm, airport winter maintenance, simulation, environmental impact

## Abstract

Airports are one of the segments within air transport and their serviceability ensures air transport efficiency. However, airports are among the largest contributors to the negative environmental impact of this kind of transport. Individual activities at airports leave a negative environmental footprint. By optimizing processes, it is possible to reduce the negative environmental impacts of these subjects. Airport winter maintenance is one of the important activities accompanying airports in geographical areas where winter season is expected due to the climate zone and weather conditions. Effective winter maintenance ensures optimal airport operation and has a significant impact on safety. However, the winter maintenance of the airport causes an excessive burden on the environment around the airports, in particular by applying de-icing agents to aircraft and movement areas, or by emissions and noise caused by equipment for snow and ice removal. The aim of the present article is to optimize the winter maintenance of the airport, namely the deployment of winter maintenance equipment with respect to meteorological conditions while maintaining the priorities for winter maintenance between individual airport areas. The aim of optimization is to achieve the saving of maintenance time and reduce the negative environmental impact of winter maintenance by effectively deploying equipment and devices.

## 1. Introduction

Transport is one of the basic attributes that ensure the modern society, economy, and living standards of people to function properly [1]. Air transport is one of the most important elements of the world transport system [1]. It is one of the fastest-growing sectors. This is evident not only by many technological changes and innovations but also by the predictions of the development of the use of passenger and freight air transport. In order to ensure the efficiency of this transport sector, it is needed that all components that enter the operating cycle in a certain way work without failures. The main components of the air transport system include airports, air traffic management systems, aircrafts and airlines [1]. All components involved in air transport constitute a very complex system.

The omission of one of the components can markedly disrupt the efficiency of the whole system operation. In addition to the benefits provided by transport systems, their adverse effects such as air pollution, emissions, noise, traffic accidents and, at present, congestion also should not be overlooked [2,3]. The negative impacts of air transport are particularly evident in the field of environment. According to ICAO, both air passenger traffic and air freight traffic are expected to more than double in the next two decades [4] and so increase its negative effects on the environment [5,6]. Despite various national and international regulatory measures, the negative environmental impact of air transport is still significant [7]. 

Airport operations, as part of the air transport system, produce 5% of the total emissions generated by air transport [8]. Correct and effective set up of activities at an airport affects its operational efficiency. Energy efficiency, ecologic efficiency, and safety are today the key factors in development [9]. Accordingly, there is an increasing need for the development of environmentally friendly and sustainable airports with minimal environmental impact [10,11].

Surrounding of airports is not only polluted by aircraft that consume large amounts of fuel. Airport logistics, which provides luggage transportation, refueling, and aircraft service, also pollutes the air. The problem of airports is high noise concentration, pollution, and congestion of transport in the surroundings [8].

Airports are also a major source of various types of waste, such as water runoff from airport surfaces containing high levels of chemicals and other toxic substances from aircraft and airport surfaces de-icing, fuel leaks, fire extinguishing foam, oil, aircraft maintenance chemicals, support equipment, etc. Measures taken to address the waste problem include the construction of sewage and wastewater treatment plants as well as drainage systems, surface and groundwater quality monitoring systems, grease separation systems, the use of biologically degradable de-icing and antifreeze agents, optimization of winter maintenance, etc. [8,12,13,14].

### Winter Maintenance of Airport

Airplanes need tire-pavement friction during taxiing, take-off, and landing. The presence of snow reduces the friction, and therefore, there is a need to understand how much friction can be expected on the different types of snow. This can be caused due to higher precipitation intensity during wet snow precipitation, or possibly because the wet snow, in contrast to slush, is a compressible material that gets compacted and fills the underlying pavement texture [15]. Pilots need accurate predictions on the quality of runway surface conditions when operating on runways disrupted by snow/ice. These predictions are typically made by friction measurements, or by expert judgments of runway inspectors [16]. Maintaining operational safety and the status of airport runways during snowfall events is a challenging issue with which many airports are grappling. Ice and snow impacts on transportation infrastructure systems add significant costs in the form of snow removal, damaged pavement and lost productivity due to travel delays. Most transport category aircraft are prohibited from operating on runways covered by untreated ice or by more than an inch of snow or slush. Hence, it is imperative that both small and large airports maintain operational status during snowfall events to support the existing operations. Conventional ice and snow removal practices are labor-intensive and bring environmental concerns, such as possible contamination of water bodies in the proximity of runway and airport pavements [17]. Snow removal is an important problem in certain countries. The main goal is to find good lower bounds on the optimal objective function value in a limited time, and we present extensive computing/processing tests comparing the obtained bounds and the times needed for the different models [18,19]. The surface of the reinforced runways must be maintained in such a condition as to ensure good friction characteristics and low rolling resistance. All impurities, such as snow, ice, standing water, mud, sand, oil, tire wear, etc., must be removed quickly and completely to minimize their accumulation in the airport’s operating areas.

If the snow cannot be removed from different parts of the airport’s movement areas at the same time, the following order should be followed for cleaning of the aerodrome:

- Runway in use
- Taxiways relevant for the runway in use
- Communications for rescue and fire services
- Check-in area
- Other areas

This order may, if necessary, be changed after an airport user’s agreement [19].

In particular, icing causes problems at airports by freezing on airport technical equipment (e.g., lighting systems), wing entering edges and other parts of aircraft, which may reduce their technical functionality. The icy surface of the runway can considerably impair braking effects as well as have a negative effect on aircraft possibility to take off and land. Its appearance on runways is a safety risk.

Technical mechanical equipment for snow removal and ice clearing from airport movement areas should be always used when the situation at the airport requires it. In the course of equipment selection, the airport operator must consider a number of factors particularly scope and type of operation, equipping of the airport by technical mechanical equipment, dimensions of airport areas, climatic conditions, and economical factors of purchase and operation of technical equipment, availability of spare parts and the possibility of repairs. The number of pieces and efficiency of technical equipment for winter maintenance is possible to determine based on the average thickness of snow layer, the quantity of snowfall during one snowfall, surface area of movement areas that need to be cleaned. The airport category gives the scope of airport equipping by technical equipment for winter maintenance and it is assigned in aeronautical information handbook.

Many researchers and studies cover winter airport maintenance. Winter maintenance studies cover different phases of winter maintenance. A significant number of studies are aimed at preventing and correctly adjusting winter maintenance preparations. In particular, the prediction of meteorological conditions [20,21], identification of different road friction conditions in the winter season and on this basis selected options for defrosting movement areas [22], measuring road temperature and optimal use of road protection materials [23,24], whether the creation, characterization, and evaluation of innovative superhydrophobic coatings on asphalt concrete surfaces for flexible icing and snow-free road applications [25]. The study [26] also deals with the ecological removal of snow from airport areas by installing a heater to prevent the use of chemical spraying.

The second group are the studies dealing with the impacts of winter maintenance on surface of movement area of an aerodrome, and the effect of the technical mechanisms for removing winter contaminants from road surface in combination with the use of de-icing agents [27] and the effect of chemical de-icing agents on the road [28].

Moreover, optimization of winter maintenance also affects managerial decisions and management of winter maintenance using human resources or support information systems [29,30].

Any airport whose geographical location and meteorological conditions predict winter season must have a winter maintenance plan in place, for example [31,32,33].

The aim of this article is to optimize the winter maintenance of the airport. Under the current conditions, processes optimization is one of the methods of regulating and reducing the negative environmental impacts of aviation. As mentioned above, airports are one of the main contributors to environmental pollution, which is also caused by the application of de-icing agents or emissions not only from aircraft but also from technical means of maintenance. Optimization of winter maintenance will also be reflected in the reduction of the negative impacts of the technical means of maintenance of winter movement areas.

The aim of the optimization is to reduce the winter maintenance time by a suitable choice of the deployment of maintenance technology. Research has been carried out with a view to reducing the negative environmental impact of airports, too. By reducing winter maintenance time, it is possible to reduce emissions from machinery and aircraft in a wait for landing. Thanks to the proposed algorithm, it is possible to optimize the deployment of the winter maintenance technique concerning meteorological conditions, while maintaining the priorities for winter maintenance between individual airport areas. By optimizing the airport winter maintenance, on the one hand, maintenance time savings are achieved, thereby making the operation of the airport more efficient and, on the other hand, reducing the negative environmental impact of winter maintenance.

The article gradually describes the inputs and outputs of the winter airport maintenance model, introduces the calculation relationships and explains the functionality of the model. Since this is a general model, the applicability of the algorithm for any airport is described.

## 2. Algorithm for Modeling of Airport Winter Maintenance

To solve the problem by algorithm means to transform input data to output data. Transformation is realized by individual steps that mean to realize in one moment only one elementary step. Both input and output data are defined by input and output conditions (properties).

The proposed algorithm is adapted to the airport model and the winter maintenance equipment so as to simulate the assumed conditions, that could occur or, more precisely, estimates the airport winter maintenance situation for real meteorological conditions.

### 2.1. Inputs and Outputs of Airport Winter Maintenance Model

The input data of the algorithm (see Appendix A) are represented by the basic parameters, which designate the airport operator. Variables are parameters, which have an influence on algorithm output and way of calculations using, procedures and functions in the algorithm. The actual airport parameters belong between the most important inputs for calculation in the algorithm. These parameters are digestedly introduced in Table 1. The algorithm is preset for the maintenance of complete airport areas.

For calculation of the model situation, the parameters of Kosice Airport, Inc. were used. These parameters are introduced in Table 2.

Another input is the type of snow according to the characteristics and its density. Snow weight is an important parameter that determines the amount and type of technical mechanical equipment used for airport winter maintenance. Snow weight also affects the performance of the equipment. The algorithm works with the assumption that there is an objectively measured value of snow height, which determines the optimal deployment of winter equipment.

The choice of winter equipment is determined as a package for which the snow removal performance is given in units (weight per unit of time). Based on these data, it is possible to continuously estimate the time for completion of work on individual airport areas. The used parameters and variables with their descriptions are described in Table 3.

Estimated time to clean the movement area *x* ∈ {*R*, *T*, *C*, *A*} of the airport:

*ETime^x^: = (QSnow × CHSnow × p^x^ × 0.01)/MP* (1)

Total estimated time to clean up airport movement areas:

*ETime: = max*_*xϵ*{__*R,T,C,A*}_{*StarTime*^*x*^ × 1/6 + *ETime^x^*} (2)

The number of parts divided by airport areas (runways):

*N*: = [*ETime^x^ × 6*] (3)

Winter maintenance on surfaces of the same type *x* is assumed to take place in parallel. We consider the dividing of airport areas in this algorithm only for the areas of runway type. In relation to the number *N*, all the upper part is selected so that the termination is at the latest in a ten-minute interval. This dividing is necessary to make the areas cleanable at 10-min intervals correspond to the real snow intensity and snow removal intensity. In the case of extreme values (snow calamity), these values could cause a disproportionate division and it is, therefore, appropriate to specify an upper limit for the variable *N*. There is no universal value because it depends on the areas of the airport itself and, of course, on the equipment used. The issue of *N* parameter limits is not addressed in this article. In the case of the real implementation of this algorithm, it would be appropriate to develop a methodology for determining the limits of the *N* parameter. However, such a procedure requires constant checking to avoid disregarding a notification (warning) that would need to confirm or refuse before the next calculation procedure. No immediate response is required for simulations, but during monitoring of real conditions is needed to react on the given notification (warnings) in real-time.

### 2.2. Calculation Relations Used in the Aalgorithm

Table 4 shows the dependence of the time required for milling snow on apron areas and the thickness of the snow layer.

The input data to the algorithm are specially set of the operational unit, selection and type of airport area. The output represents the time of snow removal from the entrance airport area and the volume of removed snow. For calculation the following relations were used:

*S = W × L* [m^2^] (4)

*V* = *S* × *h* × 0.01 [m^3^] (5)

*ρ* = *m*/*V* [kg × m^−3^] (6)

where:
*S* [m^2^]surface of the area*W* [m]width of the area*L* [m]length of the area*h* [cm]height of snow*ρ* [kg *×* m^−3^]density of snow*v* [km *×* h^−1^]drive speed of unit*m* [kg]weight of snow*V* [m^3^]volume of snow*w* [kg *×* h^−1^]snow thrower performance per hour

The values shown in Table 4 are also modifiable for other equipment involved in airport winter maintenance. Details have been described in articles [28,29]. The relevance of the technical data that ensures airport winter maintenance is on the airport side. These values (from which the intensity of snow removal is determined) represent the main input that affects the accuracy of the calculations with an estimate of the reference values.

### 2.3. Description of the Model with the Explanation of Functionalities

The airport winter maintenance simulation algorithm described below for a 24-h cycle begins with the designation of variables and all inputs. The next step is to set the timer control variables and CCSC to value 1 to ensure the number of repetitions exactly as indicated in the simulation timeout (update). The cycles in this simulation are numbered from the first (value 1) to the last in the 24-h cycle (value 144). The zero time is not taken into account. Auxiliary variables are set to zero. This is done to make sure that if the program runs more than once (more 24-h cycles), the old values are stored inside and not affect the variables, which could lead to incorrect results.

The height of snow (HSnow) is value, which is taken from the sensor placed in the airport space or from the values database for simulation. Subsequently, it is put into the first element of the current field (i.e., the current height of snow, CHSnow [15,35]), because it is inserted in computing relations. If the simulation cycle has not yet been started, then the first element must be counted from the sensor or the simulation database.

Moreover, for correct calculations, it is necessary to use the right relations and count data input. It is important to define the individual parameters and variables that define the nature of the airport and at the same time provide input for time estimation.

The main cycle of the algorithm is simulated within 24 h. This cycle begins at row 006 and ends at row 041. The control variable in this cycle is the timer. This variable specifies the timer, the current time at the 24-h cycle. There are 144 repetitions of our cycle at the 24h cycle. Each of these repetitions lasts 10 min, which, when multiplied by 144, gives a 24-h result.

The first branching determines whether or not current snow height from the sensor placed on the movement areas is less than 2 mm (see rows 008 to 011). If true, the program will count actual snow height from the sensor and fit it into the current element of scope CHSnow (see rows 009 till 010). If this condition is not met, the program will continue to use the calculated CHSnow from the previous cycle. This should be done because if it starts to snow intensely immediately after the check, the current snow level value in the program will remain unchanged for 10 min until the cycle is complete. During these 10 min it may be affected by so much snow that the intensity of snow is greater than 2 mm in 10 min, or the actual snow height is greater than 2 mm. It can actually be accomplished, but the program would consider it false and for that reason would skip to the end of the cycle without running the cleaning process. To prevent this, every time the schedule says that the mechanism has cleaned landing areas, it must be confirmed by the sensor first to ensure a safe landing. If the sensor confirms the calculations, the condition is not met, and the program continues normally.

Secondary branching (see rows 012 to 039) represents the alone decision concerning cleaning/not cleaning the movement areas. If the current snow height or the current snow intensity is above the 2 mm tolerance, the process of the movement area cleaning starts.

If not, the program tells us that there is no snow on the movement areas and the aircraft can land safely. At the end of the whole algorithm (see rows 044 to 046) there is a sequence block that tells the user about the current snow level and the time of cleaning the airport movement areas. In addition, there is a delay block that commands the program to wait 10 min before proceeding to the next cycle (see row 048). The whole algorithm ends at the end of 10 min cycle together with the ending of all programs [19,34].

## 3. Analysis of Model Adaptation to Optional Airport

The algorithm shown in Appendix A and described above is a generalized version of the algorithms that were developed for Kosice airport. The main generalization is the possibility to set the number of runways, the number of taxiways, connecting routes as well as the number of aprons of the simulated airport. This option enables creating the model of any airport that has such areas. Depending on the number of areas, the dimensions are also entered and the area they occupy is calculated. Since the entry is separate for each item, it is also possible to enter for example runways of different dimensions.

Since winter maintenance takes into account the priorities for winter maintenance between individual airport areas, these areas can be cleaned separately. However, there is a limitation that areas of the same type are maintained in a non-parallel manner, that is, maintenance begins on one surface and after completion, it passes to the other and the next surface in order and ends when all surfaces have passed cleaned. Parallel maintenance is also permitted, but this would have to be assigned in row 009 a value that does not correspond to the sum of the individual areas, but only the value that corresponds to the maximum area of the same type. However, this possibility is not solved by the presented algorithm.

It is also possible to realize parallel maintenance of airport areas by running the introduced algorithm in parallel several times so as to capture all areas on which the equipment came out at the same time and started parallel maintenance of airport areas. In this case, it might be advisable to program an add-on module that would coordinate the parallel execution of the presented algorithm and also summarize their results into one summary result.

## 4. Results

The model is based on the optimal deployment of maintenance tools depending on snow height and density. It provides an estimate for making the airport available for take-off and landing of aircraft, taking into account selected maintenance techniques, metrological conditions, and priorities for cleaning the airport movement areas.

The general nature of the algorithm allows setting the number of runways, taxiways, connecting routes and also the number of aprons of the simulated airport. This option allows creating a model of any airport that has such areas. Depending on the number of areas, the dimensions are entered and the covered area is calculated. Since the entry is separate for each item, it is also possible to enter the take-off and landing area of different dimensions.

The simulation of the change in meteorological conditions, which was created in the original algorithm for the Kosice Airport, Inc. is also a part of this algorithm. This option is created only for the runway’s areas as they are the most important part of the airport and also essential from the safety point of view. For other airport parts, general cleaning is always assumed before the cycle should be repeated. If the parameter corresponding to the snow height is updated accordingly, partial maintenance simulation is also possible for other areas.

The defined algorithm is universally applicable to any airport that meets the input assumptions explained in Table 1 and Table 2.

Since it is a theoretical model tested with theoretical data, it does not offer specific empirical results and its testing with real data was not possible due to the unavailability of a suitable software application. However, this theoretical model forms a suitable basis of the upcoming software support.

## 5. Discussion

Thanks to the presented algorithm, it is possible to simulate the situation at the airport using simulation meteorological data and the technique available for estimation to make the airport available for landing and takeoffs.

Optimization can reduce the time of winter maintenance, allowing aircraft to spend less time waiting to land under adverse weather conditions and release fewer pollutants and emissions. At the same time, if the estimate is unfavorable, it is possible to react in time and prepare the technique to minimize the time of winter maintenance of the airport.

The algorithm can also be used for on-line monitoring of the situation with real data when it can continuously estimate the status of the airport maintenance and thus the possibility of a real-time reaction to the meteorological situation and the status of the airport maintenance.

The model limitation can be seen in the following areas.

Since winter maintenance takes into account the priorities for winter maintenance between individual airport areas, these areas can be cleaned separately. However, there is a limitation that areas of the same type are maintained in a non-parallel manner. That is, maintenance begins on one area and after completion, it passes to second and other areas in seriate and ends when all areas have passed cleaned. Parallel maintenance would have to adjust the given algorithm calculation accordingly. Another limitation in the presented algorithm is the choice of performance class of maintenance equipment. It is possible to select only the same performance class for all areas. This deficiency can be easily eliminated in this algorithm. However, such a dynamic choice requires a deeper analysis of the proceeding of the winter maintenance equipment. It is also possible to realize this without interfering with the algorithm, but with the parallel execution of the algorithm for multiple areas and also with different performance class of the equipment used on the given area, which is cleaned in parallel.

Optimizing winter airport maintenance has many benefits in terms of airport environmental impact (aircraft emissions and technical maintenance), economic impact (airport usability), and, last but not least, airport safety.

The time, economics and resulting fuel and emission savings are dependent on the volume of air traffic at a particular airport. As the volume of air traffic increases, airport management seeks to reduce winter maintenance time and, on this basis, sometimes inefficiently uses winter maintenance resources (deploying more resources than needed).

The presented theoretical model is an input for the upcoming software user-friendly application usable for airport management when planning winter airport maintenance.

In the real simulation, it is expected to save tens of minutes of heavy equipment operation (engine power 300 kW) with consumption of 200 g of fuel per kW, which is about 50 L per hour of operation. Precise savings are the result of efficient use of the model.

## 6. Conclusions

The submitted algorithm for simulation of a 24-h cycle for winter maintenance on a predefined airport represents only the generalized version of the already existing algorithm with the changed methodology of time estimates calculation. The approximation function was replaced by direct function, which was needed for calculation of estimates for any airport with different airport areas. Despite partial restrictions, this algorithm is a very good base for research in this field and possibly even broader generalization of this algorithm, with more selection of options, would eventually lead to increased dynamics of calculation. It would also be advisable in the future to try to automatize the way of equipment selection for winter maintenance according to current meteorological conditions and other conditions, which can be added into this system by some external intervention. Here, however, the process of generalization may not be easy to solve considering very high variability of technical equipment for winter maintenance at individual airports. To solve this problem, it would be necessary to create a system of approximations for the choice of individual technical sets for winter airport maintenance.

By making the airport winter maintenance more efficient, it is possible to achieve maintenance time savings, thus increasing the airport’s operating time. Another benefit is to reduce the negative environmental impact of winter airport maintenance through efficient planning, the use of staff time, and technical mechanisms. Due to the efficient use of mechanization means, it is possible to reduce, for example, their emissions around the airport.

## Figures and Tables

**Table 1:** Basic dimensions of the airport.

Parts of Airport Movement Areas	Dimensions of Airport Movement Areas	The Surface Area of Airport Movement Areas
**Runways (1, …, *k*_1_)**	*W_j_^R^* × *L_j_^R^* [m]	*S_j_^R^* [m^2^]
**Taxiways (1, …, *k*_2_)**	*W_j_^T^* × *L_j_^T^* [m]	*S_j_^T^* [m^2^]
**Connecting routes (1, …, *k*_3_)**	*W_j_^C^* × *L_j_^C^* [m]	*S_j_^C^* [m^2^]
**Apron (1, …, *k*_4_)**	*W_j_^A^* × *L_j_^A^* [m]	*S_j_^A^* [m^2^]
**Total area**		*S* [m^2^]

**Table 2:** Basic dimensions of Kosice Airport, Inc. [30].

Parts of Airport Movement Areas	Dimensions of Airport Movement Areas	The Surface Area of Airport Movement Areas
**Runway**	3000 × 45 [m]	135,000 [m^2^]
**Taxiways**	3000 × 23 [m]	69,000 [m^2^]
**Connecting route 1**	300 × 23 [m]	6900 [m^2^]
**Connecting route 2**	300 × 23 [m]	6900 [m^2^]
**Apron**	200 × 100 [m]	20,000 [m^2^]
**Total area**		237,800 [m^2^]

**Table 3:** All inputs, parameters, variables, and their description.

Title	Type	Description
**HSC**	Matrix of real numbers	Snow height in different parts: This variable represents the height of the snow in a given part and time
**ISnow**	Series of real numbers	Snow Intensity: Input data obtained from the sensor. How many cm of snow come in 10 min
**CHSnow**	Series of real numbers	Actual snow height: This value is a trend, the average snow depth in the whole airport. It is the average snow height for each part of the airport.
**ETime**	Series of real numbers	Estimated time: Variable representing the expected time to clean up airport areas
**ETime^x^**	Series of real numbers	Estimated time: The variable representing the expected cleaning time of the area *x* ∈ {*R*, *T*, *C*, *A*} of the airport
**QSnow**	Series of real numbers	Snow quality: The input from the sensor is the current snow quality based on the classification
**HSnow**	Real number	Snow height: input from Sensor of current snow depth
**MP**	Series of real numbers	Machinery performance: Based on snow properties, the employee will determine the appropriate machinery
**Timer**	Integer	Timer: A control variable that evaluates in 10 min cycles for 24 h. It monitors the situation at the airport for 24 h.
**CCSC**	Integer	Currently cleaned parts: Variable representing the currently cleaned parts
**NSc**	Integer	Area part number: A variable specifying the order of the cleaned part in cycles
**N**	Integer	Amount of parts: A variable representing the number of parts and the need to divide the cleaned area in order to keep the cleaning time limit
**SSnow**	Real number	Snow amount: Auxiliary variable containing height of snow in all parts of the cleaned airport area
**Hr**	Integer	Hour: Conversion of an auxiliary variable for the number of cycles to hours
**Min**	Integer	Minute: Conversion of an auxiliary variable for the number of cycles to minutes in a given hour
***k*** **_1_**	Integer	Number: Number of runways
***k*** **_2_**	Integer	Number: Number of taxiways
***k*** **_3_**	Integer	Number: Number connecting routes
***k*** **_4_**	Integer	Number: Number check-in and parking areas
***W_j_^x^***	Real number	Width: See the definition in the table (Table 1) for the parameter *x* ∈ {*R*, *T*, *C*, *A*}
***L_j_^x^***	Real number	Length: See the definition in the table (Table 1) for the parameter *x* ∈ {*R*, *T*, *C*, *A*}
***S_j_^x^***	Real number	Surface: See table definition (Table 1) for parameter *x* ∈ {*R*, *T*, *C*, *A*}
***StartTime^x^***	integer	Starting time: The number of the time cycle in which the winter maintenance of the *x* ∈ {*R*, *T*, *C*, *A*} started

**Table 4:** The time needed for milling of snow [15,34].

Apron Area	The Thickness of the Snow Layer	Total Weight of Snow	Volume of Snow	The Net Time Needed to Mill the Snow
10,000 m^2^	2.5 cm	100,000 kg	250 m^3^	1.5 min
	5 cm	200,000 kg	500 m^3^	3 min
	10 cm	400,000 kg	1000 m^3^	6 min
	15 cm	600,000 kg	1500 m^3^	10 min
	25 cm	1,000,000 kg	2500 m^3^	15 min
16,000 m^2^	2.5 cm	160,000 kg	400 m^3^	2.5 min
	5 cm	320,000 kg	800 m^3^	5 min
	10 cm	640,000 kg	1600 m^3^	10 min
	15 cm	960,000 kg	2400 m^3^	13 min
	25 cm	1,600,000 kg	4000 m^3^	22 min
25,000 m^2^	2.5 cm	250,000 kg	625 m^3^	3.5 min
	5 cm	500,000 kg	1250 m^3^	7 min
	10 cm	1,000,000 kg	2500 m^3^	15 min
	15 cm	1,500,000 kg	3750 m^3^	23 min
	25 cm	2,500,000 kg	6250 m^3^	35 min
50,000 m^2^	2.5 cm	500,000 kg	1250 m^3^	7 min
	5 cm	1,000,000 kg	2500 m^3^	15 min
	10 cm	2,000,000 kg	5000 m^3^	30 min
	15 cm	3,000,000 kg	7500 m^3^	45 min
	25 cm	5,000,000 kg	12,500 m^3^	75 min
Hourly power of the snowblower in tonnes			4400 t	
Constant snow specific weight at −4 °C			400 kg·m^−3^	

## Appendix A

The algorithm of airport winter maintenance optimization.
